# A composite PET-matrix patch enhances tendon regeneration and tendon-to-bone integration for bridging repair of the chronic massive rotator cuff tears in a rabbit model

**DOI:** 10.1093/rb/rbae061

**Published:** 2024-06-19

**Authors:** Yuyan Na, Hao Jue, Tian Xia, Moxin Li, Xiaoao Xue, Yinghui Hua

**Affiliations:** Department of Sports Medicine, Sports Medicine Institute of Fudan University, Huashan Hospital, Fudan University, Shanghai 200040, China; Department of Sports Medicine, Sports Medicine Institute of Fudan University, Huashan Hospital, Fudan University, Shanghai 200040, China; Department of Sports Medicine, Sports Medicine Institute of Fudan University, Huashan Hospital, Fudan University, Shanghai 200040, China; Department of Sports Medicine, Sports Medicine Institute of Fudan University, Huashan Hospital, Fudan University, Shanghai 200040, China; Department of Sports Medicine, Sports Medicine Institute of Fudan University, Huashan Hospital, Fudan University, Shanghai 200040, China; Department of Sports Medicine, Sports Medicine Institute of Fudan University, Huashan Hospital, Fudan University, Shanghai 200040, China

**Keywords:** massive rotator cuff tears, bridging repair, patch, PET, decellularized dermal matrix

## Abstract

In recent years, bridging repair has emerged as an effective approach for the treatment of massive rotator cuff tears (MRCTs). The objective of this study was to develop a composite patch that combines superior mechanical strength and biocompatibility and evaluate its potential for enhancing the outcomes of bridging repair for MRCTs. The composite patch, referred to as the PET-matrix patch (PM), was fabricated by immersing a plain-woven PET patch in decellularized matrix gel and utilizing the freeze-drying technique. The results demonstrated that the PM has reliable mechanical properties, with a maximum failure load of up to 480 N. The decellularized matrix sponge (DMS), present on the surface of the PM, displayed a loose and porous structure, with an average pore size of 62.51 μm and a porosity of 95.43%. *In vitro* experiments showed significant elongation of tenocytes on the DMS, with cells spanning across multiple pores and extending multiple protrusions as observed on SEM images. In contrast, tenocytes on the PET patch appeared smaller in size and lacked significant elongation. Additionally, the DMS facilitated the proliferation, migration and differentiation of tenocytes. In a rabbit model of chronic MRCTs, the PM group showed superior outcomes compared to the PET group at 4, 8 and 12 weeks after bridging repair. The PM group displayed significantly higher tendon maturing score, larger collagen diameter in the regenerated tendon and improved tendon-to-bone healing scores compared to the PET group (*P *<* *0.05). Moreover, the maximum failure load of the tendon–bone complex in the PM group was significantly higher than that in the PET group (*P *<* *0.05). In summary, the PM possesses reliable mechanical properties and excellent cytocompatibility, which can significantly improve the outcomes of bridging repair for chronic MRCTs in rabbits. Therefore, it holds great potential for clinical applications.

## Graphical Abstract

**Figure:**
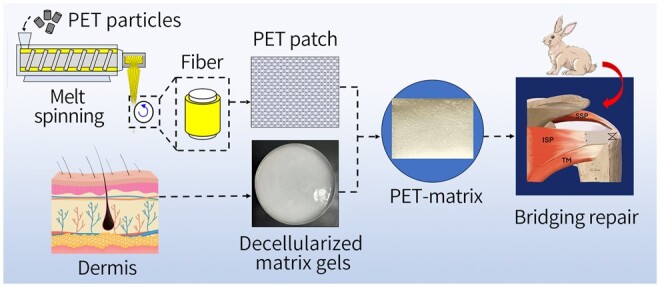

## Introduction

Rotator cuff tears are a prevalent shoulder condition, of which massive rotator cuff tears (MRCTs) have always been a clinical treatment challenge [1–3]. The negative impact of MRCTs on shoulder mobility severely compromises patients’ overall quality of life. Achieving anatomical restoration of MRCTs is challenging, often resulting in a high retear rate (79%) after repair [2]. However, with the development of arthroscopic surgical techniques, various surgical strategies have been proposed and applied to treat MRCTs, such as superior capsular reconstruction, bridging repair, tendon transfer and reverse shoulder arthroplasty, among others [4–8].

In recent years, with the development of tissue engineering, bridging repair has been favored by some surgeons. A review systematically analyzed 15 clinical studies of bridging repairs of MRCTs and found that bridging was effective in improving patients’ functional shoulder scores and was superior to nonanatomic repairs in terms of functional outcomes [9]. The bridging repair is an anatomic repair by bridging the tendon stump to the footprint of the greater tuberosity through a graft. Therefore, this technique is effective in restoring the normal contractile function of the rotator cuff muscles, which in turn alleviates muscle degeneration, such as muscle atrophy and fatty infiltration [10]. It is worth noting that severe muscle degeneration has been identified as a significant risk factor for retear after MRCTs repair [11, 12].

Currently, there is a wide range of grafts used in bridging repair, including autografts, allografts, xenografts and synthetic materials [9, 13–16]. However, it remains unclear which graft yields superior repair outcomes. Clinical studies on bridging repair for MRCTs have primarily consisted of case series using a single graft, lacking comparative analysis [13–16]. Therefore, in our preliminary research, we investigated the efficacy of autologous fascia lata (FL), acellular dermal matrix (ADM) and polyethylene terephthalate (PET) patch as bridging patches for chronic MRCTs in rabbits. The results demonstrated successful repair of MRCTs using all three grafts. However, some distinctions emerged. The PET group was weaker than the biologic grafts in promoting tendon and fibrocartilage regeneration. On the other hand, the ADM group had the lowest mechanical properties for the healed tendon–bone complex. Consequently, the combination of the easily accessible PET patch with biologic acellular matrix is expected to improve the bridging outcomes.

In the present study, microscale PET fibers were first woven into a mesh structure as warp and weft yarns. The bovine dermis was then decellularized and pulverized to obtain a matrix gel. Subsequently, the PET woven was immersed in the matrix gel and subjected to freeze-drying, resulting in a composite patch with a porous and sponge-like surface. The cytocompatibility of the decellularized matrix sponge (DMS) and PET patch was then evaluated *in vitro*. Furthermore, the *in vivo* healing of bridging for chronic MRCTs in a rabbit model using the composite PET-matrix patch (PM) was assessed histologically and biomechanically. We hypothesized that: (i) the DMS would have better cytocompatibility compared with PET patch, and (ii) the PM patch would improve the histological and biomechanical outcomes of bridging for chronic MRCTs in rabbits.

## Materials and methods

### PET fibers fabrication and patch weaving

The PET particles (Zhejiang Hengyi Petrochemical Co., Ltd) were initially placed in a vacuum rotary drum oven (JM-500ZDGX, Shanghai Jinma Electro-optical Technology Research Institute) and dried at 80°C for 4 h to remove moisture. Following that, the PET was pre-crystallized by vacuum drying at 100°C for 4 h for spinning. Round PET primary fibers were then prepared using a melt spinning equipment (produced by Shanghai Jinwei Chemical Fiber Machinery Manufacturing Co., Ltd) with a 36-hole round spinneret. Finally, the obtained primary fibers were thermally drawn at 70°C and thermally set at 100°C to obtain fiber tows. Considering the excellent mechanical properties of plain fabrics, which can better meet the mechanical requirements of the bridging patch, a plain weave was chosen as the organizational structure. The warp and weft densities of the patch were designed as 260 fibers/10 cm and 260 fibers/10 cm, respectively. The patches were woven on a semi-automatic prototype machine of Y208W, following the processes of yarn winding, hemming, reed drawing and warping.

### Decellularized dermal matrix gels preparation

After the removal of bovine dermal accessory structures such as subcutaneous fat, muscle and hair, the dermis was treated with a 2–5-N sodium hydroxide solution for 10–60 min and repeated three times. Subsequently, it was washed with a 1–5% Tween solution for 30 min. The harvested decellularized dermal matrix was then immersed in a 1000-ml 0.2% aqueous acetic acid solution for 48 h, followed by crushing to achieve an emulsion. Finally, the emulsion was filtered through a 100-mesh sieve to obtain a homogeneous lower clear liquid, referred to as the decellularized dermal matrix gel.

### Fabrication of the PM patch

The process of combining PET patches with decellularized matrix gel is illustrated in Graphical Abstract. The PET patches were first fixed in the mold, and then the decellularized matrix gel was injected into the mold and left for 10 min. The mold was then placed in a vacuum chamber at −0.1 Bar for 30 min to enhance the infiltration of the gel into the patch. After that, the mold was transferred to a freeze dryer at −5°C for 4 h of pre-freezing. The temperature of the freeze dryer was then set to −20°C, the vacuum was turned on and the PM patch was obtained after 48 h of freeze drying. The core of the PM patch is made of a PET fabric, while the surface is composed of a DMS.

### DNA and collagen content assay of DMS

The harvested DMS were then subjected to DNA and hydroxyproline content testing, with the control group consisting of fresh dermis without decellularization. DNA extraction from both groups was performed using a DNA extraction kit (TIANamp Genomic DNA Kit), and the DNA content was quantified using a spectrophotometer. The collagen content of the two groups was measured using a hydroxyproline assay kit (A030-2-1, Nanjing Jianjian).

### Ectopic implantation

To evaluate the degradation rate of the implanted DMS, a total of five male rabbits weighing 2.5 kg were selected for ectopic implantation. The rabbits were administered general anesthesia using Zoletil 50, a combination of tiletamine and zolazepam, at a dosage of 0.3 ml/kg via intramuscular injection. After shaving and sterilizing the dorsal skin, a longitudinal incision of ∼15 mm in length was carefully made. Subsequently, the subcutaneous fascia was bluntly dissected to create a pocket-like structure. A round DMS, measuring 10 mm in diameter, was precisely inserted into the pocket. To ensure stability and easy identification, a No. 4-0 Ethibond suture (Ethicon) was threaded through the edge of the sponge. The pocket was securely closed afterwards. At specific postoperative time intervals of 1, 3, 7, 14 and 28 days, the implanted DMS were retrieved for HE stains.

### Scanning electron microscope

Scanning electron microscope (SEM) (Hitachi SU8010, Japan) was used to observe the microscopic morphology of PET fabric and DMS. The 5 × 5 mm sponges were fixed in 2.5% glutaraldehyde for 12 h, followed by a series of procedures including rinsing, post-fixation, gradient alcohol dehydration and critical point drying. The samples were then coated with gold spray and placed on observation side up for imaging. In the captured images, the diameters of 30 pores were measured to determine the average sponge pore diameter. The porosity of the sponge was quantified by employing Image J software to calculate the ratio of the total pore area to the total area seen in the electron microscope images.

After the tenocytes were cultured on PET patch and DMS for 1, 3 and 7 days, the cell adhesion and growth were observed using SEM (ZEISS Gemin300). The procedure was the same as the method described earlier, after fixation in 2.5% glutaraldehyde for 12 h, rinsing, post-fixation, gradient alcohol dehydration, critical point drying and gold-spray coating were performed sequentially and then SEM images were taken.

### *In vitro* experiment

#### Isolation and culture of rat tenocytes

Tenocytes were extracted from rat Achilles tendons according to the methods described previously [17, 18]. After harvesting the Achilles tendon, the surrounding connective tissue and adipose were removed, followed by rinsing with PBS. The tissue was then cut into pieces and incubated with 3 mg/ml collagenase at 37°C for 1 h. To ensure optimal digestion, the tissue was blown every 10 min. After digestion, an equal volume of complete medium was added to stop the digestion. The mixture was filtered through a 100-μm sieve, transferred and subjected to centrifugation at 300 g/1000 rpm for 10 min. The supernatant was discarded, and the cells were resuspended in complete medium before being transferred to cell culture flasks. Passage 3 cells were selected for subsequent experiments.

#### Cell proliferation and cell viability staining

The patches were cut into circular shapes of 5 mm and 2 cm diameters, utilizing appropriate cutting molds. Subsequently, the patches were subjected to sterilization using ethylene oxide. Prior to cell implantation, the patches were soaked in DMEM overnight.

The effect of different patches (DMS and PET) on cell proliferation was assessed using the CCK8 assay and DNA content test. Tenocytes were seeded onto 5 mm patches and samples were collected after 1, 3 and 7 days of incubation using 96-well plates. The optical density values were measured at 450 nm using a microplate reader. The DNA extraction kit (TIANamp Genomic DNA Kit) was then used to extract DNA from the cells cultured for 1, 3 and 7 days, respectively. Subsequently, the extracted DNA was analyzed using a spectrophotometer.

The effect of different patches on tenocytes viability was evaluated using a live-dead staining assay. After 1, 3 and 7 days of culture, the staining solution was added to 12-well plates and incubated at 37°C with 5% CO_2_ for 15 min. Subsequently, the samples were observed and captured using a fluorescence microscope (Olympus Corporation, Tokyo, Japan). Differences in the number of live cells from the fluorescent images were counted using Image J software.

#### Transwell migration assay

The migratory activity of tenocytes was assessed using Transwell migration assay (Corning) to evaluate the effect of different patches. The DMS or PET patch was positioned at the bottom of a culture dish. Subsequently, the cell density was adjusted to 1 × 10^5^/ml, and 0.2 ml of cell suspension was inoculated into the Transwell chamber. The cells were incubated for 8–16 h at 37°C with 5% CO_2_. After incubation, paraformaldehyde was added to fix the cells. The cells were then washed with PBS and stained with 0.5% crystal violet solution. Following staining, the cells were washed with PBS, dried and observed under a light microscope (DM4000B, Leica).

#### RNA isolation and quantitative PCR

To compare the expression levels of tendonogenic-related genes in tenocytes cultured on different patches, reverse transcription-polymerase chain reaction (RT-QPCR) was employed. After 7 days of culture, total RNA was extracted using TRIzol reagent (Invitrogen). Quantitative PCR was carried out using the SYBR Green QPCR Master Mix (TakaRa) and the Light Cycler instrument (ABI Stepone plus). The expression levels of type I collagen (COL1A1), scleraxis (SCX), tenascin C (TNC) and tenomodulin (TNMD) genes were evaluated. The primer sequences used in this study can be found in Supplementary Table S1. Three experimental replicates were conducted for each quantitative PCR analysis.

### *In vivo* experiment

#### Animal study design

The animal experimental protocol was approved by the Institutional Animal Ethics Committee (DHUEC-NSFC-2021-39). This work has been reported in accordance with the ARRIVE guidelines (Animals in Research: Reporting In Vivo Experiments) [19]. A total of 81 male New Zealand rabbits, averaging 3.0 ± 0.5 kg in weight, were enrolled in this study. All rabbits were randomly assigned to the negative control (NC) group, PM repair group and PET repair group. To simulate MRCTs in humans, chronic and massive defects of the infraspinatus tendon were established in rabbits as previously described [20–22]. After general anesthesia, the skin was shaved and sterilized, and then a longitudinal incision of ∼30 mm in length was made on the posterior-lateral aspect of the right shoulder. The infraspinatus tendon was visible after the skin incision, and the overlying deltoid muscle was incised. The infraspinatus tendon was detached from its anatomical footprint, and the cartilage within the footprint was completely removed using a No. 15 blade. To mimic an irreparable MRCTs model, an 8-mm length of the infraspinatus tendon was removed, with verification that the stump could not be pulled back into the footprint. To facilitate identification of the stump, a No. 4-0 Ethibond suture (Ethicon) was passed through the tendon stump and knotted. The deltoid muscle was then sutured, and the skin incision was closed. Postoperatively, intramuscular administration of Penicillin G was continued for three consecutive days.

#### Bridging repair

**Figure 1.**
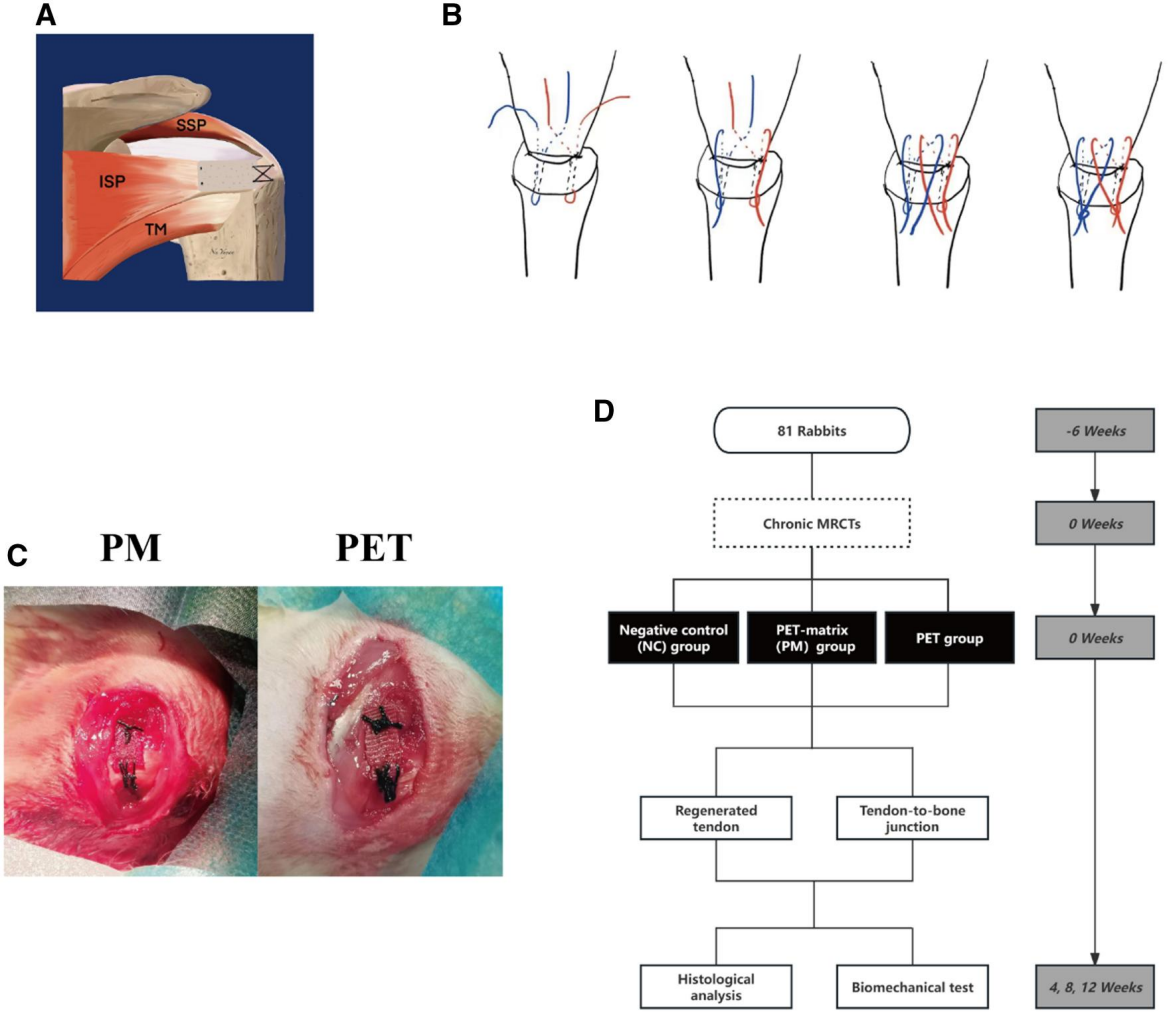
Schematic representations of the bridging repair techniques for rabbit MRCTs. (**A**) a schematic diagram of bridging repair of infraspinatus tendon. (**B**) Suture bridge technique is used in the fixation of the patch to the footprint. (**C**) Completion of bridging using a PM or PET patch. (**D**) The animal experimental process. SSP, supraspinatus; ISP, infraspinatus; TM, teres minor; PM, the composite PET-matrix patch; PET, polyethylene terephthalate.

Preliminary study by our group have revealed that significant muscle atrophy and fat infiltration occurs at 6 weeks after infraspinatus tenotomy. Therefore, 6 weeks after the initial surgery, all rabbits underwent bridging repair of MRCTs under general anesthesia. After the skin was shaved and sterilized, an incision was made along the original incision on the right shoulder, and regenerated fibrovascular tissue was seen to reattach to the footprint. Removal of the regenerated fibrovascular tissue along the marked sutures. After the NC group performed the above operations, the incision was closed directly. Subsequently, the patch was sutured to the upper surface of the tendon stump through two mattress sutures (No. 2-0 Ethibond sutures). For fixation of the patch to the bone, two parallel bone channels were drilled with 1.0 mm K-wire from the footprint to the lateral aspect of the greater tuberosity. Then, the patch was secured to the footprint using a suture bridge method (Figure 1B). In this method, a suture needle with an open loop was initially used to guide the ends of two No. 2-0 Ethibond sutures through the bone channels. Subsequently, the ends of sutures were crossed over the patch and knotted together with the loop left on the lateral aspect of the greater tuberosity for fixation. The patches were bridged to the bone as shown in Figure 1C, with one end sutured to the stump, while the other end firmly anchored to the humeral footprint. Finally, the deltoid muscle and skin were sutured layer by layer. Postoperatively, penicillin G was administered intramuscularly once daily for three consecutive days.

Rabbits were euthanized by injection of an overdose of Zoletil 50 at 4, 8, 12 weeks after bridging. After *in vivo* and *ex vivo* observation of tendon-to-bone healing, histologic evaluation (4 per group) and biomechanical testing (5 per group) were performed.

#### Transmission electron microscopy evaluation

Transmission electron microscopy (TEM) was employed to assess the diameter of collagen fibers in the regenerated tendon. Fresh tendons were immediately trimmed into a 3 × 1 mm^2^ size and subsequently fixed using 2.5% glutaraldehyde at 4°C for 24 h. After PBS washing, post-fixation, dehydration, filtration and embedding, the tissues were ultrathin sectioned and stained. Finally, the tissues were transferred to copper grids and observed under TEM (Hitachi HT7800, Japan). The diameter distribution of the regenerated collagen fibers was determined using Image J software and Origin 2021, and at least 500 fibers were measured for each sample.

### Histological analysis

All histological samples were fixed in 4% paraformaldehyde for 48 h, followed by decalcification with 10% EDTA for 3–4 weeks. After confirming satisfactory decalcification through needle puncture, the samples were dehydrated and embedded in paraffin. Tissue sections with a thickness of 4 μm were obtained along the horizontal plane of the infraspinatus tendon-bone complex. Subsequently, sections were subjected to hematoxylin and eosin (HE) staining, Safranin O/Fast green (SF) staining and Picrosirius red (PR) staining to evaluate the histological morphology of the regenerated tendons and tendon–bone interface, as well as the regeneration of fibrocartilage and the deposition and maturation of regenerated collagen. In particular, the PR-stained sections underwent further evaluation using polarized light microscopy (Nikon Eclipse E800, Japan) to determine the content of type I and III collagen in the regenerated tendons [23]. The maturity of the regenerated tendons was semi-quantitatively evaluated using the Modified Tendon Healing Evaluation (MTHE) system (Supplementary Table S2) [24, 25]. To assess tendon-to-bone healing, the histologic scoring system introduced by Jang et al was utilized (Supplementary Table S3) [20, 26]. In the SF-stained sections, abundant accumulation of Safranin around the sutures and PET fibers, which may lead to inaccuracies in quantitative evaluation of metachromasia ratio. Therefore, we manually selected the regions of metachromasia at the tendon–bone interface (using 3D CaseViewer) and calculated the area to evaluate the extent of fibrocartilage regeneration. All section scoring was independently performed by two experienced researchers (Hao Jue and Tian Xia) in a blinded manner, with respect to time points and groups.

### Biomechanical testing

Biomechanical testing was conducted using an electronic universal testing machine (UTM4304, SUNS, China) on the PET patch, the PM patch and tendon–bone complex. Longitudinal stretching (*n* = 3) was performed on long strips of the PET and PM patch (50 × 10 mm) with an initial distance of 10 mm between the fixtures and a stretching speed of 5 mm/min. Biomechanical tests were performed on the healed tendon–bone complex at 4, 8 and 12 weeks after the repair. To ensure that the bone was not crushed during tensile testing, the humerus was immobilized using polyvinyl chloride tubing containing hardened dental powder [22]. Muscle tissue surrounding the tendon was scraped off using a knife, and then the tendon was wrapped in a layer of gauze to prevent slipping during stretch testing. Stretching was performed along the direction of force on the infraspinatus, with the tendon stretching direction perpendicular to the humerus. The overall design of the stretching device is shown in Figure 9A. The initial distance between the tendon fixture and the bone was 10 mm, and the stretching speed was set at 5 mm/min. During the test, when the rising load curve suddenly showed a significant drop, indicating that micro-rupture had already occurred in the complex. Finally, the original values of the load–deformation curves were recorded.

### Statistical analysis

The required sample size for this experiment was calculated based on the reported biomechanical test data on rabbit infraspinatus bridging repairs. To detect a statistically significant difference in maximum failure load among the groups, a minimum of five rabbits in each group was deemed necessary [20]. All quantitative data were presented as mean ± SD. Group differences were compared using Student’s *t*-test for two-group comparisons and one-way ANOVA for three-group comparisons. Statistical analysis and graphical representation were conducted using Graphpad Prism 8.0, with a significance level set at *P* < 0.05.

## Results

### Characterization of the PET patch and DMS

**Figure 2.**
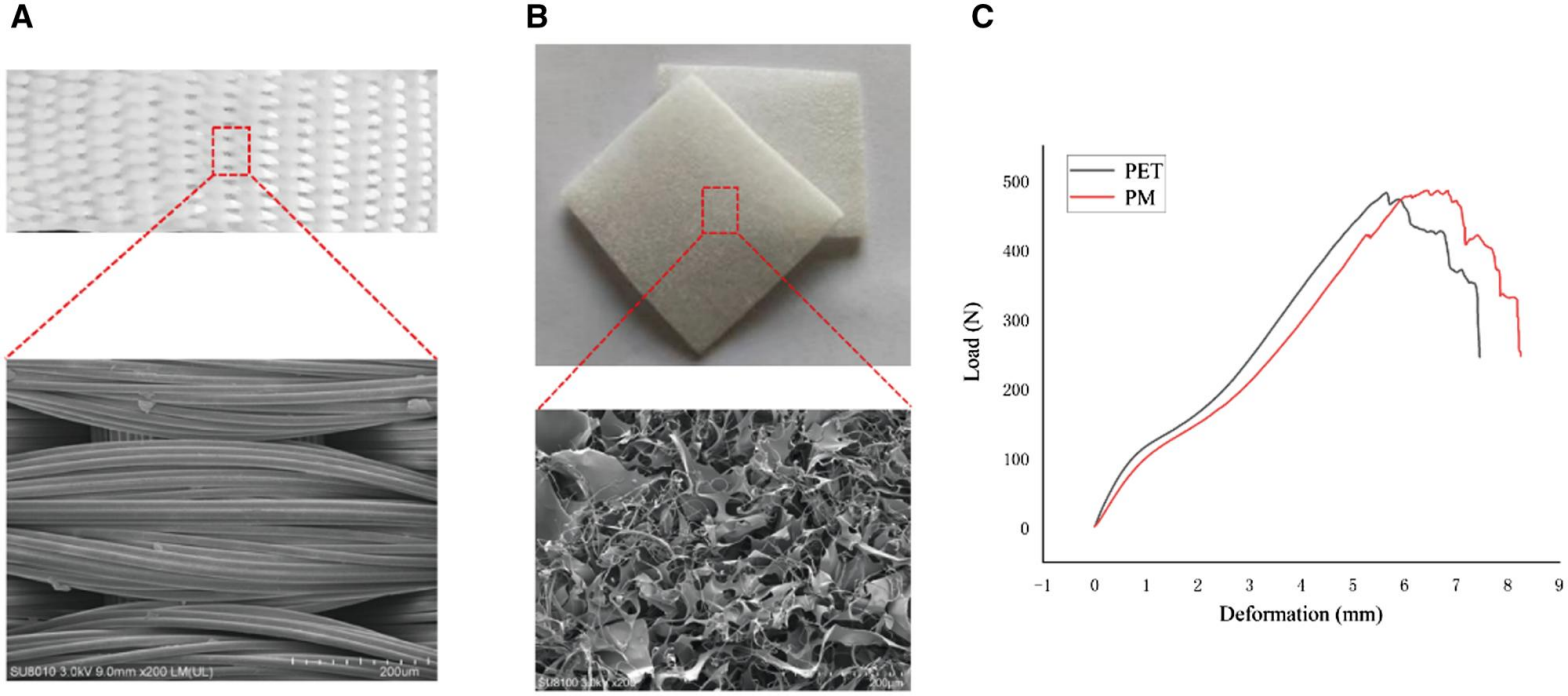
Characterization of the composite PM patch. (**A**) Gross and SEM images of the PET woven fabric located in the core layer of the composite PM patch. (**B**) Gross and SEM images of the DMS located on the surface of the composite PM patch. (**C**) Load–deformation curve of the PET and PM patch tensile testing. PM, the composite PET-matrix patch; SEM, scanning electron microscope; DMS, decellularized matrix sponge; PET, polyethylene terephthalate.

Figure 2A and B showed gross and SEM images of the PET woven and DMS. The PET fibers displayed a dense and orderly arrangement, while the DMS showed a loose and porous structure. The average pore diameter of the DMS was measured to be 62.51 μm, with a porosity of 95.43%. Subsequently, tensile tests were performed on both the PET and PM patches. The results revealed that both patches exhibited a remarkable tensile strength exceeding 480 N (Figure 2C), satisfying the mechanical requirements for bridging repair (the normal tensile strength of the infraspinatus tendon–bone complex in rabbits ranged between 80 and 160 N; the normal tensile strength of human supraspinatus tendon–bone complex ranged from 250 to 400 N) [27]. Importantly, it was demonstrated that the composite of decellularized matrix did not compromise the mechanical properties of the PET patch.

### Histologic observation of DMS and ectopic implantation results

**Figure 3.**
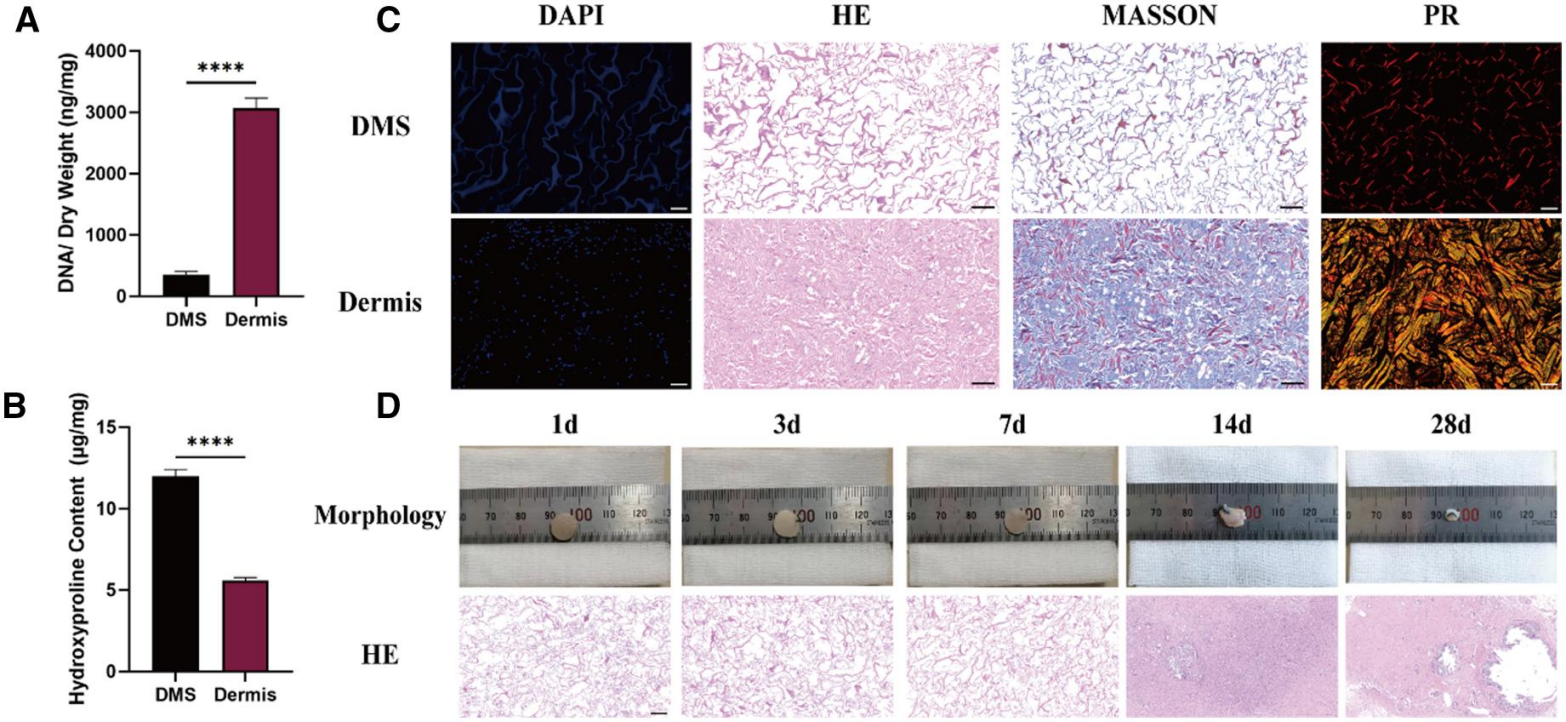
DMS characterization and ectopic implantation result. (**A**) DNA content before and after decellularization. (**B**) Hydroxyproline content before and after decellularization. (**C**) Histologic observation of DMS and dermis (DAPI, HE, Masson, PR). (**D**) The results of ectopic implantation of DMS included the observation of DMS residual volume, and HE staining to assess the structural degradation of the sponge at 1, 3, 7, 14 and 28 days after implantation. DMS, decellularized matrix sponge; DAPI, 4′,6-diamidino-2-phenylindole; HE, hematoxylin and eosin; PR, picrosirius red.

Figure 3A illustrates the changes in DNA content before and after decellularization of the dermis. It is evident that the DNA content significantly decreased following decellularization. Figure 3B demonstrated that the DMS had a higher hydroxyproline content, indicating a higher collagen concentration per unit weight. The results of DAPI staining further confirmed the significantly lower DNA content in the DMS. HE stains revealed a sparse and porous structure in the DMS, which is conducive to regenerative cell growth. Additionally, both MASSON and PR staining demonstrated that the DMS were abundant in collagen fibers (Figure 3C).

The sponges were subsequently implanted subcutaneously into the dorsal region of rabbits. At specific time points (Days 1, 3, 7, 14 and 28), HE stains were performed on the implanted sponges to assess the degradation rate. Gross images revealed a gradual degradation of the sponges during the implantation period, with almost complete disappearance observed by Day 28 (Figure 3D). HE stains revealed a substantial infiltration of inflammatory cells surrounding the residual sponge at 14 days, whereas the number of infiltrated inflammatory cells decreased significantly by Day 28.

### Cytocompatibility analysis

**Figure 4.**
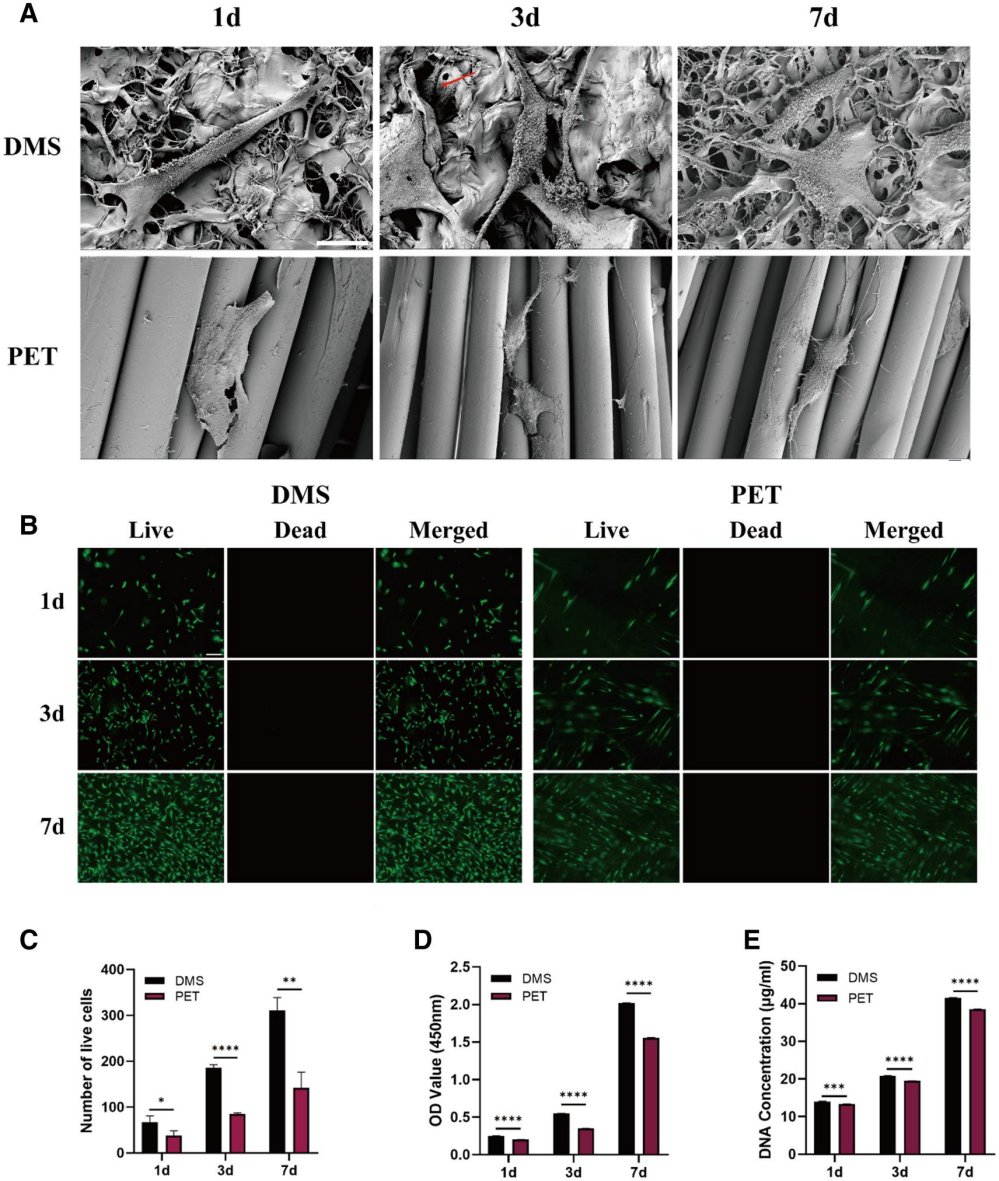
The cytocompatibility evaluation of the DMS and PET patch. (**A**) Scanning electron micrographs of tenocytes adhesion and growth on both patches, with the red arrows indicating tenocytes extending into the interior of the sponge. The scale bar equals 20 μm. (**B**) Live-dead staining analysis of tenocytes incubated on both patches for 1, 3 and 7 days. The scale bar equals 200 μm. (**C**) Number of live cells analyzed from the fluorescent images of live and dead staining. (**D**) Results of CCK8 analysis of tenocytes incubated on both patches for 1, 3 and 7 days. (**E**) DNA content of tenocytes incubated on both patches for 1, 3, and 7 days. *P <* *0.05. **P <* *0.01. ***P <* *0.001. ****P <* *0.0001. DMS, decellularized matrix sponge; PET, polyethylene terephthalate.

After seeding tenocytes onto the DMS and PET woven, the biocompatibility of the two materials was qualitatively and quantitatively evaluated after 1, 3 and 7 days of incubation. Initially, SEM was employed to observe the adhesion and growth of tenocytes on the different patches. On the DMS, tenocytes exhibited significant elongation, spanning across multiple pores. Additionally, some cells appeared to expand and extend multiple protrusions. Notably, tenocytes growing into the interior of the sponge (indicated by red arrows) were observed in the SEM images on day 3. In contrast, tenocytes on the PET patch appeared smaller in size and did not show significant elongation (Figure 4A).

The results from cell viability staining revealed a significant increase in the number of live cells in the DMS group compared to the PET group (*P* < 0.05) (Figure 4C). Moreover, cells in the DMS displayed a more spindle-like morphology, whereas those in the PET group appeared more rounded (Figure 4B). CCK8 analysis and DNA content assay revealed a gradual increase in cell proliferation activity and cell count with the extension of culture. Notably, the DMS showed significantly higher proliferation activity and cell count compared to the PET group (*P* < 0.05) (Figure 4D and E). These findings suggested that the DMS demonstrated superior cytocompatibility for tenocytes.

**Figure 5.**
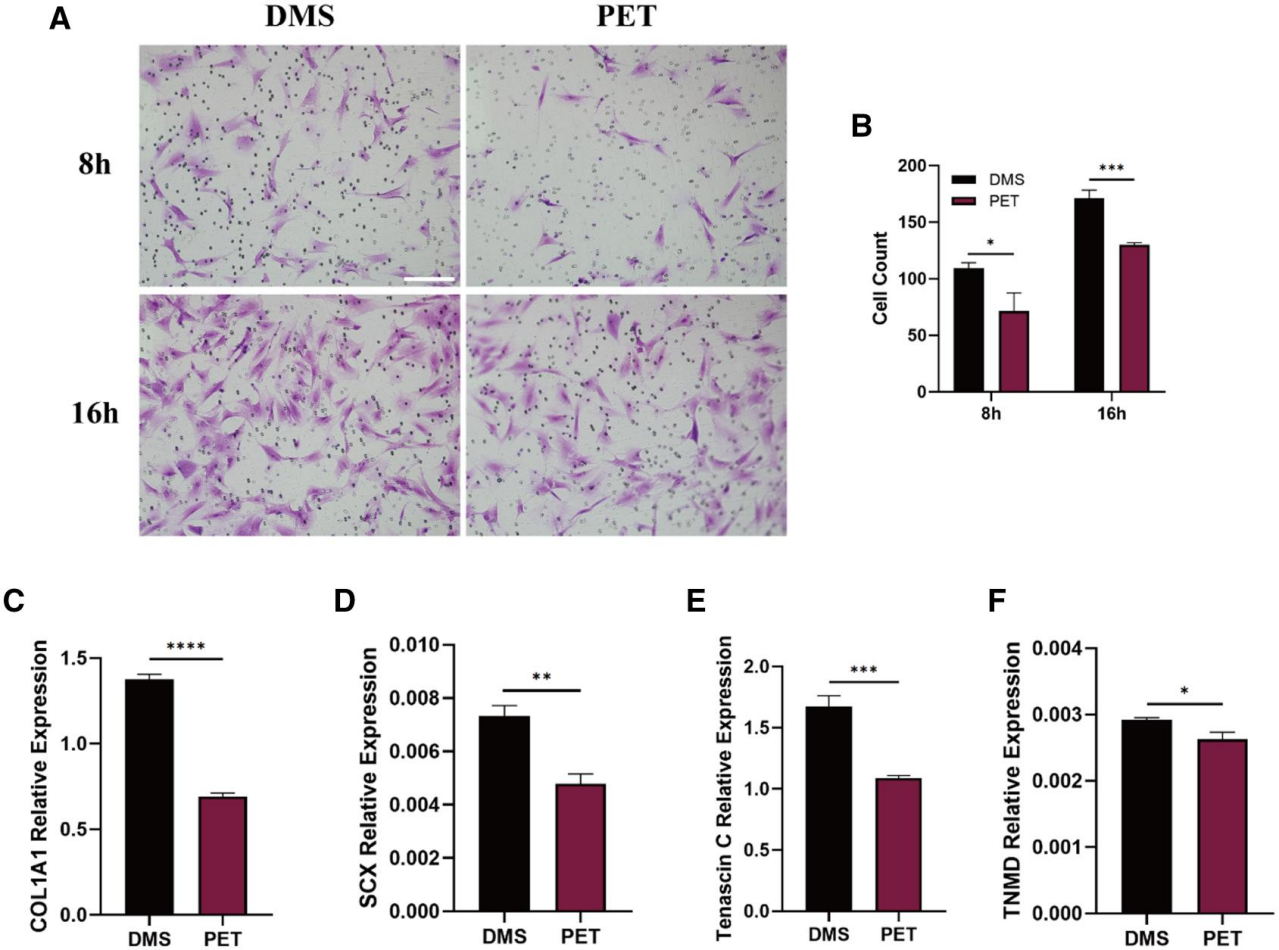
Effects of DMS and PET patch on tenocytes migration and differentiation. (**A**) Crystal violet staining of migrated tenocytes after incubation for 8 and 16 h. The scale bar equals 150 μm. (**B**) Quantitative assessment of the migrated tenocytes. (**C**–**F**) Expression of tendonogenic-related genes in tenocytes incubated for 7 days on the DMS and PET patch. *P <* *0.05. **P <* *0.01. ***P <* *0.001. ****P <* *0.0001. DMS, decellularized matrix sponge; PET, polyethylene terephthalate.

Subsequently, a transwell migration assay was used to evaluate whether the DMS could facilitate cell migration. Crystalline violet staining results demonstrated that after 8 and 16 h of culture, the migration of cells in the compartment was significantly greater in the DMS group compared to the PET group (*P* < 0.05) (Figure 5A and B). Moreover, the differences in the expression of tendonogenic-related genes in tenocytes cultured with different patches were determined by RT-PCR. As shown in Figure 5C–F, following 7 days of incubation, the DMS group displayed significantly higher expression levels of tendonogenic-related genes (COL1A1, SCX, TNC and TNMD) compared to the PET group (*P* < 0.05). These findings indicated that the DMS can effectively promote the tendonogenic differentiation of cells.

### Evaluation of *in vivo* bridging repair

#### Gross assessment of tendon-bone healing

Observations of *in vivo* and *ex vivo* specimens showed that no patch rupture, infection or tearing of the tendon-to-bone junction occurred in PM and PET groups (Supplementary Figure S1).

#### Histologic evaluation of tendon regeneration

**Figure 6.**
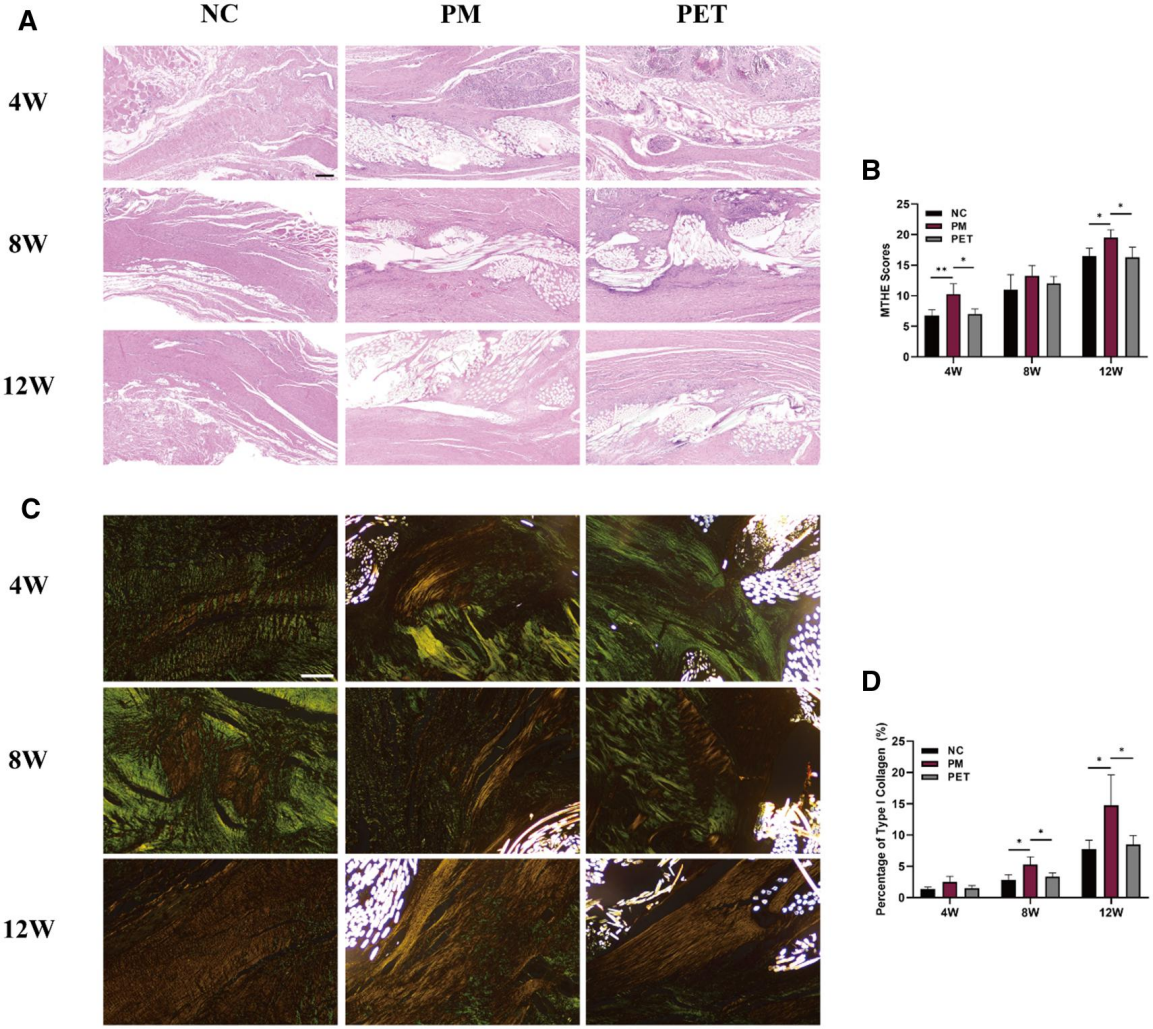
Histological analysis of the regenerated tendon substance. (**A**) HE staining images of the regenerated tendons in NC, PM and PET groups at 4, 8 and 12 weeks after repair. The scale bar equals 200 μm. (**B**) The MTHE scores for the regenerated tendon. (**C**) PR staining images of the regenerated tendons in NC, PM and PET groups at 4, 8 and 12 weeks after repair. The scale bar equals 200 μm. (**D**) Percentage of type I collagen (a percentage of the yellow pixels of each image) of the regenerated tendon in PR staining. *P <* *0.05. **P <* *0.01. W, weeks; HE, hematoxylin and eosin; PR, picrosirius red; NC, negative control; PM, the composite PET-matrix patch; PET, polyethylene terephthalate; MTHE, the modified tendon histological evaluation.

At 4 weeks after repair, all groups showed a noticeable infiltration of inflammatory cells, accompanied by significant vascular proliferation within the regenerated tendons. The regenerated collagen fibers were loose and disorganized. At this stage, the MTHE score in the PM group (10.25 ± 1.71) was significantly higher than that in the NC group (6.75 ± 0.96) and PET group (7.00 ± 0.82) (*P* < 0.05) (Figure 6A and B). At 8 weeks, there was a remarkable reduction in the number of infiltrated inflammatory cells around the grafts. Moreover, a substantial number of regenerated collagen fibers had grown into the interior of the PET patch, with a more orderly arrangement of cells and collagen fibers compared to the 4-week stage. At 12 weeks, the number of cells within the regenerated tendon significantly decreased, while the proportion of long spindle-shaped cells increased. The cells and fibers displayed a more orderly arrangement. At this time, the PM group (19.50 ± 1.29) displayed a significantly higher MTHE score compared to the NC group (16.50 ± 1.29) and PET group (16.25 ± 1.71) (*P* < 0.05) (Figure 6B).

Then, the regenerated tendons were subjected to PR staining and observed under polarized light microscopy, where yellow and green colors in the tendon represented type I and type III collagen, respectively (Figure 6C). The relative content of type I collagen in the regenerated tendons was calculated based on the percentage of yellow pixels. The results revealed that as the repair time extended, the proportion of type I collagen in both groups of regenerated tendons gradually increased. At 8- and 12-weeks post-operation, the PM group showed a significantly higher proportion of type I collagen compared to the NC and PET group (*P* < 0.05) (Figure 6D).

**Figure 7.**
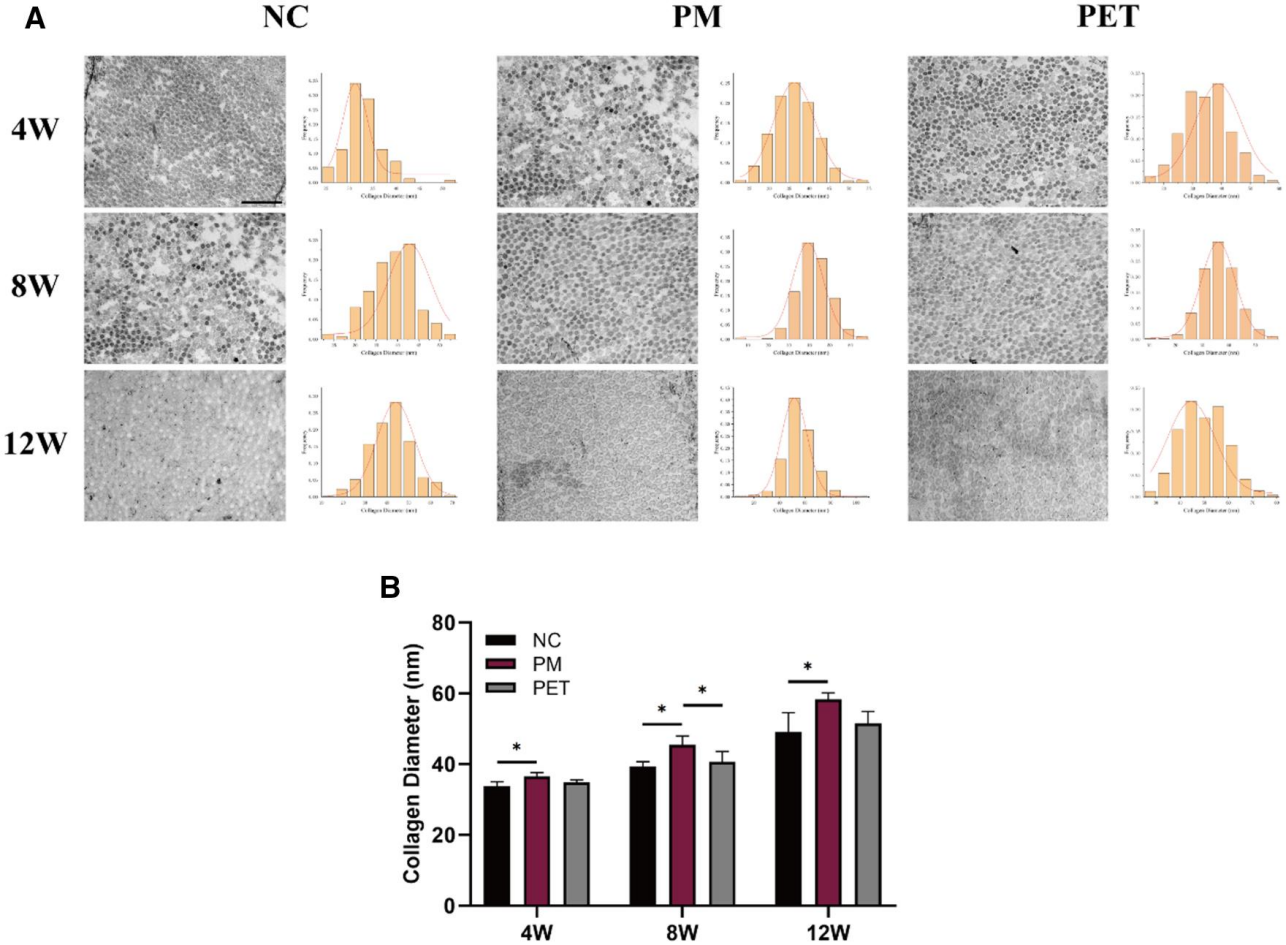
TEM images of the regenerated tendon. (**A**) TEM images and diameter distribution of the regenerated collagen fibers in NC, PM and PET groups at 4, 8 and 12 weeks after repair. The scale bar equals 500 nm. (**B**) Quantitative analysis of collagen fiber diameter in both groups. *P <* *0.05. W, weeks; TEM, transmission electron microscopy; NC, negative control; PM, the composite PET-matrix patch; PET, polyethylene terephthalate.

The diameter of collagen fibers in the regenerated tendons was determined using TEM, allowing for comparison among the three groups. In both groups, the overall distribution of regenerated collagen fibers was uniform (Figure 7A). At 4, 8 and 12 weeks post-repair, the diameter of collagen fibers in the PM group was significantly larger than that in the NC group (*P* < 0.05) (Figure 7B). At 8 weeks after repair, the PM group showed a significantly larger diameter of regenerated collagen fiber compared to the PET group (*P* < 0.05) (Figure 7B).

#### Histologic evaluation of tendon-to-bone healing

**Figure 8.**
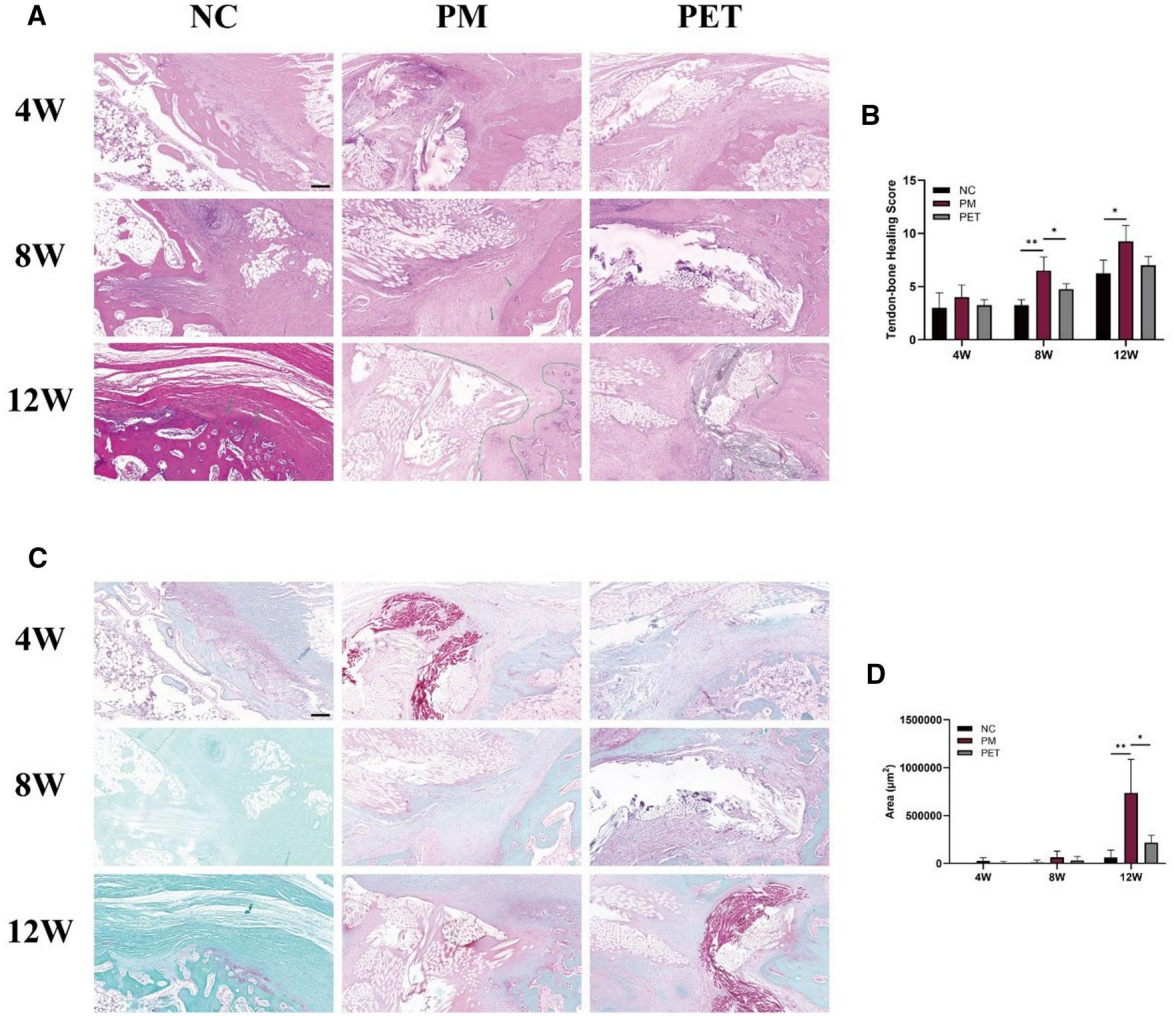
Morphologic observation of tendon-to-bone healing and assessment of fibrocartilage regeneration. (**A**) HE staining images of the tendon–bone interface in NC, PM and PET groups at 4, 8 and 12 weeks after repair. The green arrows indicate regenerated fibrocartilage. The scale bar equals 200 μm. (**B**) Tendon-to-bone healing histologic score from HE staining. (**C**) Fibrocartilage regeneration of the interface was assessed by SF staining. The scale bar equals 200 μm. (**D**) Quantifying the area of fibrocartilage regeneration at the interface in all groups. *P <* *0.05. W, weeks; HE, hematoxylin and eosin; SF, Safranin-O/fast green; NC, negative control; PM, the composite PET-matrix patch; PET, polyethylene terephthalate.

The tendon-to-bone healing score was primarily focused on assessing the presence of regenerated fibrocartilage and the extent of fibrocartilage coverage. At 4 weeks post-repair, it was challenging to observe the regenerated fibrocartilage at the tendon–bone interface in all groups. Instead, the area was predominantly occupied by infiltrated inflammatory cells and hyperplastic fibrovascular tissue, resulting in a distinct tissue transition at the interface (Figure 8A). At 8 weeks, a small amount of regenerated fibrocartilage could be observed in some fields (indicated by green arrows). At this stage, the tendon–bone healing score was significantly higher in the PM group (6.50 ± 1.29) compared to the NC group (3.25 ± 0.50) and PET group (4.75 ± 0.50) (*P* < 0.05) (Figure 8B). At 12 weeks, the amount of regenerated fibrocartilage at the interface gradually increased in all groups. Tissue transition at the interface became indistinct. At this time, the tendon–bone healing score in the PM group (9.25 ± 1.50) was significantly higher than that in the NC group (6.25 ± 1.26) (*P* < 0.05) (Figure 8B).

Furthermore, the results of SF staining revealed that the area of fibrocartilage metachromasia was significantly greater in the PM group compared to the NC group and PET group at 12 weeks postoperatively (*P* < 0.05) (Figure 8C and D). This indicated that the incorporation of DMS could facilitate fibrocartilage regeneration.

#### Biomechanical tests

**Figure 9.**
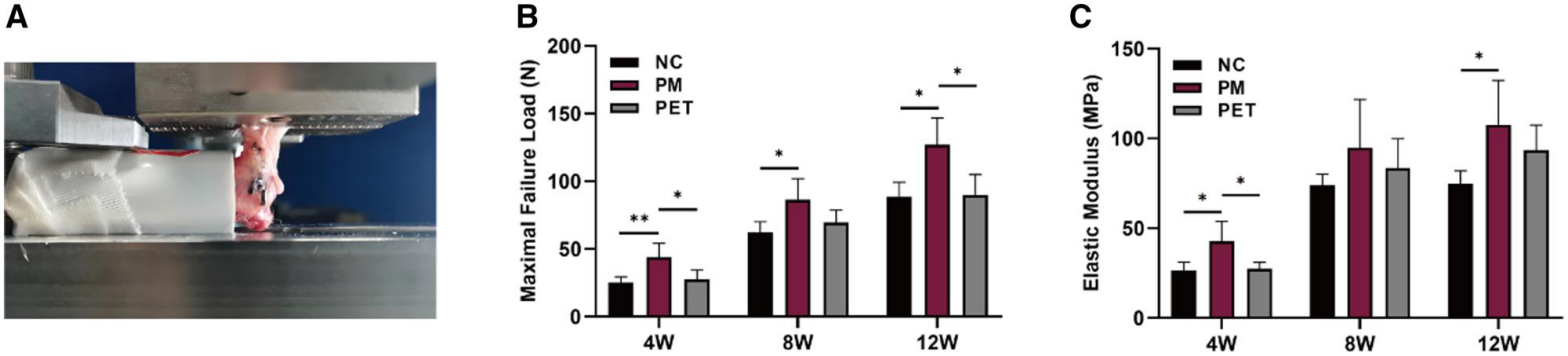
Biomechanical test results of the tendon-bone complexes. (**A**) Sample fixation and stretching device for biomechanical testing. Comparison of the maximal failure load (**B**) and the elastic modulus (**C**) in NC, PM and PET groups at 4, 8 and 12 weeks after repair. *P <* *0.05. W, weeks; NC, negative control; PM, the composite PET-matrix patch; PET, polyethylene terephthalate.

At 4, 8 and 12 weeks after bridging repair, the tendon–bone complex was subjected to biomechanical testing. The results showed that both the maximum failure load and elastic modulus of the tendon–bone complexes increased significantly over time, indicating a gradual enhancement of tendon–bone healing. At 4, 8 and 12 weeks after repair, the PM group showed a significantly higher maximum failure load compared to the NC group (*P* < 0.05) (Figure 9B). And, at 4 and 12 weeks postoperatively, the maximum failure load was significantly higher in the PM group compared to the PET group (4 weeks: 43.89 ± 10.28 vs. 27.66 ± 6.74 N, *P* < 0.05) (12 weeks: 125.10 ± 21.47 vs. 89.93 ± 15.07 N, *P* < 0.05) (Figure 9B). In addition, at 4 and 12 weeks postoperatively, the PM group showed a significantly higher elastic modulus compared to the NC group (*P* < 0.05) (Figure 9C). And, at 4 weeks after repair, the elastic modulus was significantly higher in the PM group (42.69 ± 11.00 MPa) than in the PET group (27.38 ± 3.65 MPa) (*P* < 0.05) (Figure 9C).

## Discussion

In this study, a composite PM patch that possessed favorable mechanical properties and biocompatibility was successfully fabricated. *In vitro* studies demonstrated that the DMS greatly enhanced the adhesion, proliferation, migration and differentiation of tenocytes compared to the PET patch. The results of bridging for chronic MRCTs in rabbits showed that the PM significantly facilitated tendon regeneration and tendon-to-bone healing compared with the PET patch. Meanwhile, the PM group had a significant advantage in the biomechanical results than the PET group.

In a long-term follow-up study on bridging repair using a PET patch for MRCTs, a retear rate of 70% was reported [28]. Synthetic PET fibers are preferred grafts due to their ease of manufacturing, cost-effectiveness and stable physicochemical and mechanical properties [29, 30]. However, PET fibers are known to have less biocompatibility compared to biological grafts, which can directly affect the outcome of bridging repairs. In this study, we aimed to address this challenge by integrating decellularized dermal matrix gel with PET patch. The resulting composite patch demonstrated its efficacy in promoting tenocytes adhesion, proliferation, migration and differentiation, leading to a significant improvement in the outcome of bridging repair for MRCTs.

The treatment of MRCTs has long been a challenge in clinical practice, leading to the emergence of numerous studies dedicated to the development of practical bridging patches [31–33]. Considering the limitations of using single materials, researchers have shifted their focus toward the development of composite materials. For example, Zheng *et al.* successfully fabricated a novel 3D collagen/silk ordered scaffold. This scaffold featured a core layer of woven silk fibers, providing mechanical strength and a surface layer of collagen sponge to enhance biocompatibility [32]. The scaffold demonstrated efficacy in promoting cell growth and tendonogenic differentiation. In a rabbit model, it also facilitated tendon regeneration when used for bridging repair of MRCTs. Clinically, Bi *et al.* [33] designed a sandwich patch that combining autologous FL with artificial ligaments. Bridging repair of MRCTs using this patch has shown significant improvements in functional outcomes for patients. Postoperative MRI assessment revealed the tendon-to-bone healing rate of 92%, demonstrating the effectiveness of this approach. Although the composite PM patch designed in the present study shares similarities with the above studies, it possesses unique advantages. First, both components of the composite patch (PET and decellularized dermal matrix) were made from clinically proven materials, ensuring its clinical therapeutic utility [9, 34–36]. Second, the decellularized dermal matrix contains a higher abundance of extracellular matrix composition compared to collagen [37]. At the same time, it avoids additional damage caused by obtaining autologous tissue. Lastly, the composite patch is designed for easy integration, facilitating surgical manipulation. When compared to the PET patch, the use of the composite patch for the bridging repair of MRCTs in rabbits leads to improved tendon regeneration, characterized by a larger diameter of collagen fibers and a higher tendon maturity score. It also increases the area of fibrocartilage regeneration at the tendon–bone interface, while enhancing the tensile strength of the tendon–bone complex.

Studies have demonstrated that the concentration of collagen influences both pore size and porosity [38]. Higher collagen concentrations lead to smaller pore sizes and lower porosity, while lower concentrations result in larger pore sizes and higher porosity [39]. However, lower collagen concentrations may compromise the structural integrity of the patch when exposed to body fluids, which can lead to collapse and damage during suture fixation. Conversely, higher collagen concentrations can lead to an overly compact fiber structure [40], which may hinder the growth of regenerative tissues. In this study, a decellularized dermal matrix gel with an optimal collagen concentration for the organism was used, without any additional adjustments made when combined with PET patches. The resulting DMS had an average pore size of 62.51 μm and a porosity of 95.43%. SEM images revealed that tenocytes on the sponge displayed significant elongation, spanning across multiple pores, while some cells appeared to expand and extend multiple protrusions. Furthermore, electron microscope images taken on the third day of culture demonstrated that tenocytes grew into the interior of the sponge. These results suggest that the DMS significantly facilitates tenocyte growth and adhesion.

In a study conducted by Ide *et al.* [37], a decellularized dermal matrix patch (GraftJacket, Wright Medical Technology Inc., Arlington, TN) was used to bridge MRCTs in rats. After 12 months postoperatively, a regenerative tendon–bone interface was observed, closely resembling the normal interface. And Kim *et al.* [41] utilized a decellularized dermal matrix patch (SureDerm, Hans Biomed Co., Daejeon, Republic of Korea) to bridge MRCTs in rabbits. At 8 weeks postoperatively, regenerative tendon displayed a structural similarity to the normal tendon. These results are consistent with our present results. In the present study, bridging repair with the PM patch in rabbits, a small amount of regenerated fibrocartilage was observed at 8 weeks. However, at 12 weeks after repair, there was a significant increase in the amount of regenerated fibrocartilage. And the maturity of regenerative tendon also significantly improved at 12 weeks after repair. In addition, the tendon-to-bone healing scores and the MTHE scores of the PM group were superior to those of the PET group. It is important to note that the DMS used in our study was derived from dermis and retained most extracellular matrix components, such as collagen, proteoglycan and elastin [37]. These extracellular matrix components are believed to play a pivotal role in promoting tendon regeneration and maturity, as supported by the results of our cellular experiments demonstrating that the DMS facilitates tenocyte proliferation, migration and differentiation. Further studies are required to investigate the specific mechanisms of fibrocartilage regeneration promoted by the DMS.

This study has the following limitations. First, the DMS used in this study was derived from the dermis. However, it is worth considering that a decellularized matrix derived from a tendon may be more beneficial in enhancing the repair outcomes of MRCTs. Second, it is important to acknowledge that there exist anatomical, functional and tissue healing differences between rabbits and humans, which may limit the generalizability of the findings. Hence, future studies should focus on validating the effectiveness of the composite patch in large-animal models of MRCTs that show physiological similarities to humans. Additionally, extending the postoperative follow-up duration in these models is necessary. While a 12-week follow-up adequately addresses tendon–bone healing in small animals, longer follow-up of 6 and 12 months after surgery are required in large animals. Despite these limitations, the developed composite patch demonstrates promising utility and effectively enhances the outcomes of MRCTs bridging repair, thereby holding significant potential for clinical applications.

## Conclusion

The present study demonstrated that the composite PM patch featured a loose and porous structure and showed reliable mechanical properties. Furthermore, the PM patch possesses excellent cytocompatibility, which was highly conducive to the adhesion, proliferation, migration and differentiation of tenocytes compared to the PET patch. Significantly, the results of bridging repair for the chronic MRCTs in rabbits displayed superior histologic and biomechanical outcomes in the PM group compared to the PET group. In summary, the composite PM patch holds great potential for clinical applications.

## Supplementary Material

rbae061_Supplementary_Data
