# Supplementary material for: A composite PET-matrix patch enhances tendon regeneration and tendon-to-bone integration for bridging repair of the chronic massive rotator cuff tears in a rabbit model
Source: Regen Biomater. 2024 Jun 19;11:rbae061. doi: 10.1093/rb/rbae061 (PMC11211210; doi:10.1093/rb/rbae061)
Supplement: rbae061_Supplementary_Data [file rbae061_supplementary_data.zip › Appendix Figure 1.docx]

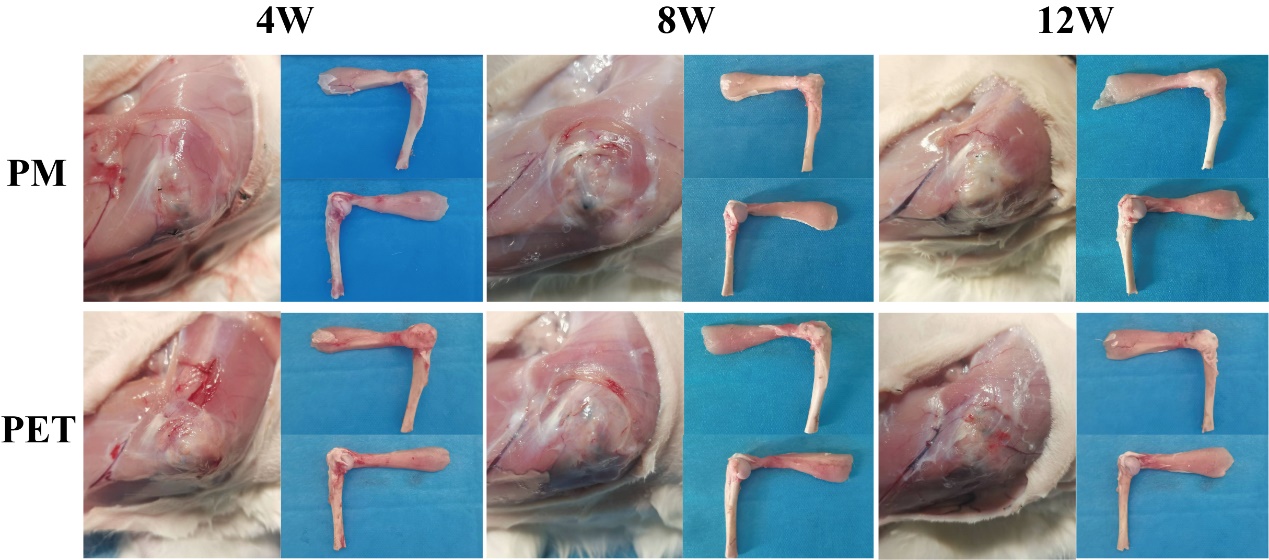


Appendix Figure 1. Gross observations of in vivo and ex vivo healing of the tendon-bone complex at 4, 8, 12 weeks after repair.
