# Supplementary material for: A composite PET-matrix patch enhances tendon regeneration and tendon-to-bone integration for bridging repair of the chronic massive rotator cuff tears in a rabbit model
Source: Regen Biomater. 2024 Jun 19;11:rbae061. doi: 10.1093/rb/rbae061 (PMC11211210; doi:10.1093/rb/rbae061)
Supplement: rbae061_Supplementary_Data [file rbae061_supplementary_data.zip › Appendix Table.docx]

Appendix Table 1 List of primer sequences used for real-time Polymerase Chain Reaction

| Genes | Forward | Reverse | Production size (bp) |
| --- | --- | --- | --- |
| COL1A1 | CTG ACT GGA AGA GCG GAG AGT A | GCT GAG TGG GGA ACA CAC A | 114 |
| TNC | CCC TGT CAG TGC CAG AGT | TCC CTC ATC TTC TTT GTT CAT A | 160 |
| SCX | AGA ACA CCC AGC CCA AAC | GAA TCG CCG TCT TTC TGT C | 87 |
| TNMD | CCT CAG CAG TGG TCT CTC AGT | TGA CAG CCC TCA CAG TTC TC | 99 |
| GAPDH | TGT TCC TAC CCC CAA TGT AT | TTC ACC ACC TTC TTG ATG TC | 87 |

Appendix Table 2 The Modified Tendon Histological Evaluation (MTHE) score for the regenerated tendon substance

| Items | Score | | | |
| --- | --- | --- | --- | --- |
|  | 0 | 1 | 2 | 3 |
| Cell density | Severely increased | Moderately increased | Slightly increased | Normal pattern |
| Rounding of nuclei | Severely rounded | Moderately rounded | Slightly rounded | Long spindle shape |
| Cell arrangement | Severely disordered | Moderately disordered | Slightly disordered | Parallel |
| Fiber density | Severely loose | Moderately loose | Slightly loose | Compacted |
| Fiber structure | Severely fragmented | Moderately fragmented | Slightly fragmented | Continuous, long fiber |
| Fiber arrangement | Severely rounded | Moderately rounded | Slightly rounded | Parallel |
| Inflammation (area infiltrated by inflammatory cells), % | ＞30 | 20-30 | 10-20 | ＜10 |
| Increased vascularity, % | ＞30 | 20-30 | 10-20 | ＜10 |
| Maximum total score | 24 | | | |

Appendix Table 3 The histologic scoring of tendon-to-bone healing

| Items |  | Score |
| --- | --- | --- |
| 1. Cellular morphologic characteristics of interface tissue |  |  |
| Fibrocartilage with mature cartilage cells comprising ≥ 50% |  | 4 |
| Fibrocartilage with mature cartilage cells comprising < 50% |  | 3 |
| Fibrous tissue with Sharpey-like fibers comprising ≥ 50% |  | 2 |
| Fibrous tissue with Sharpey-like fibers comprising < 50% |  | 1 |
| Only fibrovascular tissue |  | 0 |
| 2. Extent of surrounding fibrocartilage tissue |  |  |
| Mostly surrounded (≥ 75%) |  | 4 |
| Moderately surrounded (50% to < 75%) |  | 3 |
| Partially surrounded (25% to < 50%) |  | 2 |
| Slightly surrounded (< 25%) |  | 1 |
| Not visible |  | 0 |
| 3. Interface tissue transition from bone to tendon |  |  |
| Mostly indistinct (≥ 75%) |  | 4 |
| Moderately indistinct (50% to < 75%) |  | 3 |
| Partially indistinct (25% to < 50%) |  | 2 |
| Continuous but slightly indistinct (< 25%) |  | 1 |
| Discontinuous |  | 0 |
| Maximum total score |  | 12 |
